# Bibliometric Analysis of Alzheimer's Disease and Depression

**DOI:** 10.2174/1570159X22666240730154834

**Published:** 2024-07-31

**Authors:** Sixin Li, Qian Zhang, Jian Liu, Nan Zhang, Xinyu Li, Ying Liu, Huiwen Qiu, Jing Li, Hui Cao

**Affiliations:** 1 Department of Psychiatry, The School of Clinical Medicine, Hunan University of Chinese Medicine, Changsha, Hunan, China;; 2 Department of Psychiatry, Brain Hospital of Hunan Province (The Second People’s Hospital of Hunan Province), Changsha, Hunan, China;; 3 Department of Neurosurgery, Xiangya Hospital, Central South University, Changsha, Hunan, China;; 4 National Clinical Research Center for Geriatric Disorders, Xiangya Hospital, Central South University, Hunan, China;; 5 Center for Medical Research and Innovation, The First Hospital, Hunan University of Chinese Medicine, Changsha, Hunan, China;; 6 College of Life Science and Technology, Huazhong University of Science and Technology, Wuhan, Hubei, 430074, P.R.China;; 7 Department of Rehabilitation, The Second Xiangya Hospital, Central South University, Changsha, Hunan, China

**Keywords:** Alzheimer’s, depression, bibliometric analysis, hotspots, CiteSpace, VOSviewer

## Abstract

**Background:**

The link between Alzheimer's disease and depression has been confirmed by clinical and epidemiological research. Therefore, our study examined the literary landscape and prevalent themes in depression-related research works on Alzheimer's disease through bibliometric analysis.

**Methods:**

Relevant literature was identified from the Web of Science core collection. Bibliometric parameters were extracted, and the major contributors were defined in terms of countries, institutions, authors, and articles using Microsoft Excel 2019 and VOSviewer. VOSviewer and CiteSpace were employed to visualize the scientific networks and seminal topics.

**Results:**

The analysis of literature utilised 10,553 articles published from 1991 until 2023. The three countries or regions with the most publications were spread across the United States, China, and England. The University of Toronto and the University of Pittsburgh were the major contributors to the institutions. Lyketsos, Constantine G., Cummings, JL were found to make outstanding contributions. Journal of Alzheimer's Disease was identified as the most productive journal. Furthermore, “Alzheimer’s”, “depression”, “dementia”, and “mild cognitive decline” were the main topics of discussion during this period.

**Limitations:**

Data were searched from a single database to become compatible with VOSviewer and CiteSpace, leading to a selection bias. Manuscripts in English were considered, leading to a language bias.

**Conclusion:**

Articles on “Alzheimer’s” and “depression” displayed an upward trend. The prevalent themes addressed were the mechanisms of depression-associated Alzheimer's disease, the identification of depression and cognitive decline in the early stages of Alzheimer's, alleviating depression and improving life quality in Alzheimer's patients and their caregivers, and diagnosing and treating neuropsychiatric symptoms in Alzheimer. Future research on these hot topics would promote understanding in this field.

## 1 INTRODUCTION

The incidence and prevalence of Alzheimer's disease and other types of dementia increased by 147.95 and 160.84%, respectively, from 1990 to 2019. The ASR of prevalence, incidence, death, and DALYs in both men and women consistently increased over the study period [1]. In America, the population with either mild cognitive or clinical Alzheimer's was 6.08 million, and this number was expected to increase to 15.0 million in 2060 [2]. Moreover, research revealed that biologically defined Alzheimer's disease was more prevalent than clinically defined probable Alzheimer's disease regardless of age [3]. Major depressive disorder (MDD) was the most prevalent mood disorder among Chinese patients because of a lifetime prevalence of 3.4% [4]. Furthermore, depressive disorder ranked 13^th^ among the top 25 leading causes of disability-adjusted life-years (DALYs) in 2019 [5]. In women with Alzheimer's disease, higher depressive vulnerability has been associated with self-criticism personality [6]. The particular genetic variations in MDD have facilitated the advancement from amnestic mild cognitive impairment to Alzheimer's disease [7]. There was a correlation between the gray matter volume (GMV) of insula atrophy and depression in AD patients, indicating a potential neural substrate in AD with depression [8].

AD and depression research has increased significantly in the last three decades. To develop diagnosis and better treatments, it is essential to keep track of the advancements and contributions of clinicians and scientists. This article aims to explore the contributions of different countries, institutions and authors and provide the foreseeable future trend in this field.

Bibliometric analysis is a statistical examination that analyzes scientific publications *via* bibliometric tools, such as CiteSpace and VOSviewer [9], to define the literature landscape and future trends [10]. A study found a positive trend in the use of bibliometrics in medicine. This trend may be due to increased productivity in bibliometric research, greater use of quantitative metrics in research evaluation, the publish-or-perish phenomenon, and the increased use of evidence-based medicine. Also, using bibliometrics in medicine can improve everyday clinical activities. This could help to increase the use of bibliometrics in healthcare [11]. Although a significant number of bibliometric analyses dealt with Alzheimer's disease and depression, respectively, none of them concentrated on the theme of depression-related manuscripts on AD. Therefore, the main aims of this article are to outline the trend of the published manuscripts, identify the popular research topics and find out which countries, institutions, authors and journals make remarkable contributions to AD with depression.

## 2 MATERIALS AND METHODS

### 2.1 Search Strategy

Data were retrieved on 10^th^ December, 2023, from the Web of Science Core Collection. The query was TS = (“Alzheimer” OR “Senile Dementia” OR “Primary Senile Degenerative Dementia” OR “Presenile Dementia”) and TS = (“depression” OR “depressive” OR “depressed” OR “Melancholia” OR “Melancholias”). Data were filtered by type of documents, including articles and reviews, and then by language, considering English. The publications were queried and screened out by Sixin Li and Qian Zhang. After that, discrepancies were sorted out by Jian Liu and Nan Zhang.

### 2.2 Data Extraction and Analytical Methods

The “full records and cited references” of these documents were extracted. VOSviewer evaluated these documents for the following data: publication years, titles, abstracts, keywords, countries/regions, institutions, authors, cited references, and journals. VOSviewer and CiteSpace, which originated from library and information science research, were frequently applied in other areas [9]. VOSviewer was an application for constructing and displaying a large bibliometric map in a graphical way [12]. CiteSpace was applied to discover emerging trends and trendy themes [13]. VOSviewer (version 1.6.20, Leiden University, Leiden, Netherlands) and Microsoft Excel 2019 (Redmond, Washington, USA) were utilized to determine the main contributors in this field, while VOSviewer and CiteSpace (Version 6.2.R6) were applied to construct scientific networks, keyword co-occurrence, and research frontier. In addition, CiteSpace was capable of illustrating keyword bursts and reference bursts. The node size in the map indicates the volume of publications, and the line shows the association among them [14].

## 3 RESULTS

### 3.1 General Data

**Fig. (1):**
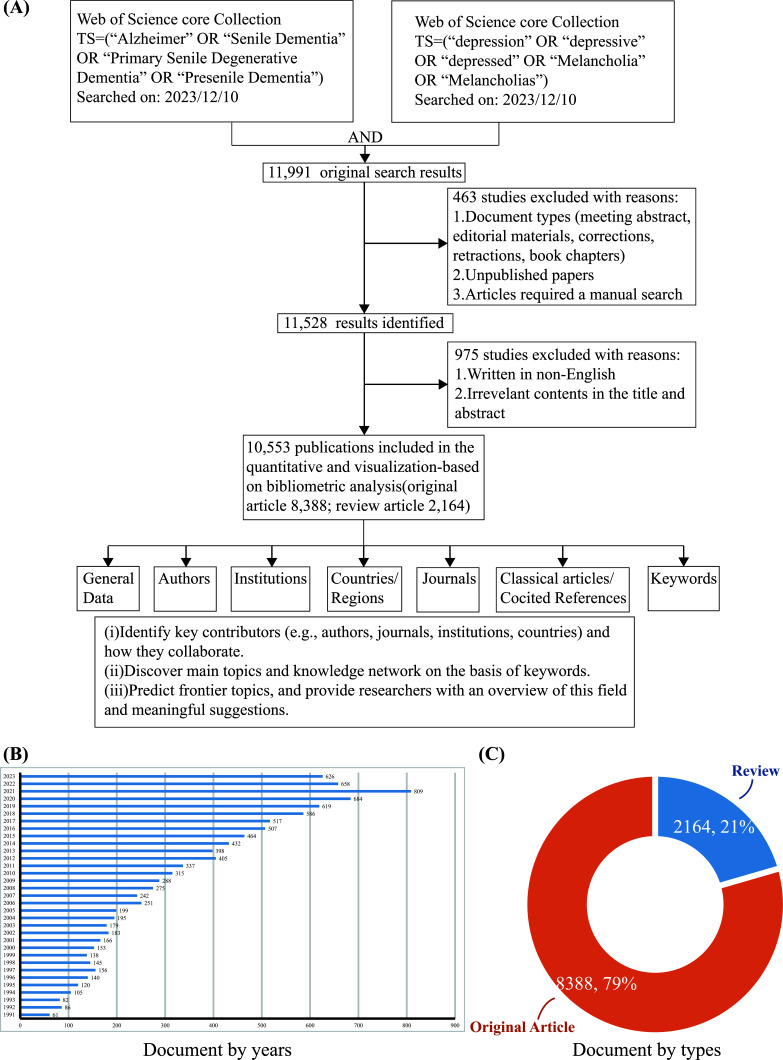
General data. (**A**) Flowchart of data screening and bibliometric analysis. Distribution of documents by year (**B**) and type (**C**).

As shown in Fig. (**1A**), the flow diagram indicates every step of the search strategy and quantity of different publications. The initial query resulted in 11,991 results. Only articles and reviews in English were considered for bibliometric analyses, leading to the final quantity of 10,553. In general, there were three phases of publication variation from 1991 to 2023, including the first phase (1991-2005), the second phase 2 (2006-2013), and the third phase (2014-2023). The publication increased slowly from 61 to 199 in the first phase, and the number in the second phase remained steady from 200 to around 400. The publication saw a remarkable increase in the third phase and reached a peak of 809 in 2021 (Fig. **1B**). The documents were dominated by original articles, with a significant percentage of 79, and the remaining were reviews, with 21% (Fig. **1C**).

### 3.2 Top Contributing Countries

Table **1** presents the top 10 countries/regions with significant contributions and notable associations. The USA was the major contributor, with 3,881 publications, followed by China with 1,003 documents. Compared with China, England had a slightly lower number of publications, accounting for 951 publications. Germany and Italy had approximately a similar publication rate, with 704 and 663 documents, respectively. Publications and CPP showed a similar pattern, with the USA ranking 1^st^ (CPP = 61.43) and England ranking 2^nd^ (CPP = 61.24). Australia (CPP = 57.85) and Canada (CPP = 55.38) rose to 3^rd^ and 4^th,^ respectively, while China dropped to the end of the list with 24.87 documents. Fig. (**2**) presents only collaborations with a minimum of 50 publications. The USA, Germany, China, Canada, and England stood out as prominent hubs with denser connections, suggesting their global academic impact in this domain and amplified cooperation. In terms of collaboration, the USA was the first-tier author with 2009 Total Link Strength (TLS) and 87 links, followed by England with 1242 TLS and 69 links.

### 3.3 Top Contributing Institutions

In Fig. (**3A**), the top 10 institutions with intensive research are displayed. The publications in the University of Toronto (publications = 210), University of Pittsburgh (publications = 199) and University of California Los Angeles (publications = 192) were above 190, accounting for the top three institutions in this field. Johns Hopkins University had slightly smaller publications, with 179. In terms of citations, the University of Pittsburgh had the highest CPP (95.3), while the University of Toronto was at the bottom of the CPP with 42.9. However, according to TLS, Johns Hopkins University ranked first (207 TLS), followed by the University of California, San Francisco (192 TLS). Fig. (**3B**) illustrates 63 institutions with at least 50 documents. University of Pittsburgh (USA), University of Toronto (Canada), University of California, Los Angeles (USA), and Johns Hopkins University (USA) were the central nodes in North America. UCL (England), King's College London (England), and Karolinska Institution (Sweden) made outstanding contributions to Europe. The results suggested that a typical region feature had characterized inter-institutional cooperation.

### 3.4 Top Contributing Authors

Table **2** presents the authors contributing to the field of AD with depression. Lyketsos, Constantine G. was the most prolific author with 84 publications, followed by Cummings, Jl with 47 documents. Herrmann, Nathan and Bennett, David A. had approximately the same publication figure, with 42 and 41, respectively. However, Folstein, Mf ranked first with 2721 co-citations, and Lyketsos, Constantine G. ranked 4^th^ with 1530 co-citations. The number of documents citing Cummings, Jl was the second highest with 2097. VOSviewer defined cooperation among researchers with a minimum of 20 publications. Ultimately, 47 authors are presented in Fig. (**4**), showing that Grant, Igor (USA) and Mausbach, Brent T. (USA) were the first-tier authors with 98 Total Link Strength (TLS).

### 3.5 Top Cited Articles

Half of the top 10 cited documents were articles (Table **3**) [15-24]. Two articles demonstrated the epidemiology of Alzheimer's disease and depression, while there were two reviews exploring the pathogenesis underlying AD and depression. Two documents proved that depression was a risk factor for AD. A review with the highest citation summarized the role of neurogenesis in AD and depression, indicating the potential part of the subgranular zone (SGZ) and the subventricular zone (SVZ) in the hippocampus [16]. An epidemiological analysis with 1679 citations, written by Murray, CJL, also dealt with the age-standardized years of life lost due to premature mortality (YLLs) rates and YLDs in this field [17]. Consistent with the document of Murray, CJL, Vos, T published the article with 1,521 citations in the Lancet and demonstrated that Alzheimer’s disease and major depressive disorder resulted in increased years lived with disability (YLDs) rates in women globally [15].

### 3.6 Top Contributing and Co-cited Journals

The top 10 contributing and cited journals are displayed in Table **4**. The most remarkable publications and TLS were found in the Journal of Alzheimer's Disease (505 publications and 3426 TLS) and the International Journal of Geriatric Psychiatry (407 publications and 3674 TLS). American Journal of Geriatric Psychiatry published 251 documents with 2958 TLS, ranking 3^rd^ in the list. However, the list of co-cited journals showed a different trend. Neurology stood out in co-citations with 20721. Likewise, both the Journal of Alzheimer's Disease and the American Journal of Geriatric Psychiatry were the 6^th^ with 8650 and the 10^th^ with 7471, respectively.

### 3.7 Analysis of Co-citation References

**Fig. (5):**
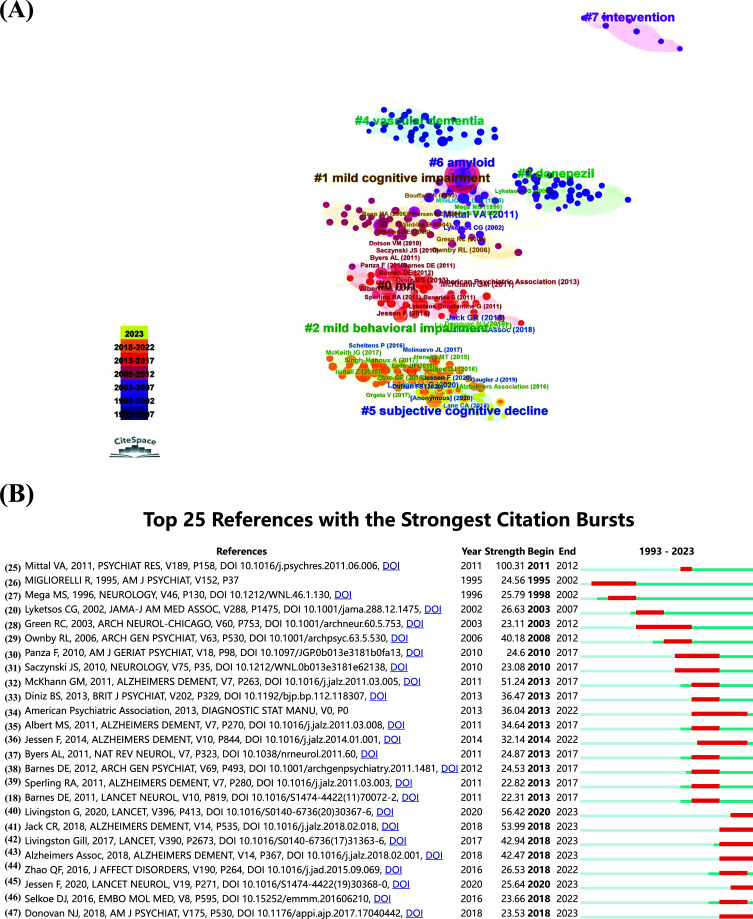
(**A**) Reference co-citation network clustered by CiteSpace. The nodes and links are colour-coded, with darker hues indicating an earlier co-citation relationship. The top 286 co-cited references are presented in a network as nodes, named according to the first author's name (year of publication). The size of each node corresponds to its citation count. The Citespace LLR algorithm automatically identified the name of the cluster. (**B**) Top 25 references with the most significant bursts. The red bar shows the duration of the burst. The intensity of the burst indicates the significance of this article to the research field.

A co-citation analysis in CiteSpace was conducted to find the knowledge network and seminal references in AD with depression. The most co-cited articles are shown in Fig. (**5A**). From 1993 to 2002, “vascular dementia”, “amyloid”, and “donepezil” were identified as hot topics in articles. Popular themes in most co-cited references from 2003 to 2012 included “intervention” and “mild cognitive impairment”, suggesting the main concern at that period. Moreover, “MRI” and “mild behavioral impairment” were the main topics in the last decade. In 2023, the primary research term changed to “subjective cognitive decline”. Moreover, a citation burst was utilized to determine seminal references. The top 25 publications with the highest citation burst were identified using CiteSpace (Fig. **5B**) [18, 20, 25-47]. Mittal VA published a manuscript with the highest strength (100.31) and demonstrated that the criteria for tic disorders is important and might affect the classification of dyskinesias in psychotic spectrum disorders [25]. The article written by Jack CR *et al*. reported the research framework and biological definition of AD and demonstrated that depression was considered the initial presentation of AD [41].

### 3.8 Analysis of Keywords

**Fig. (6):**
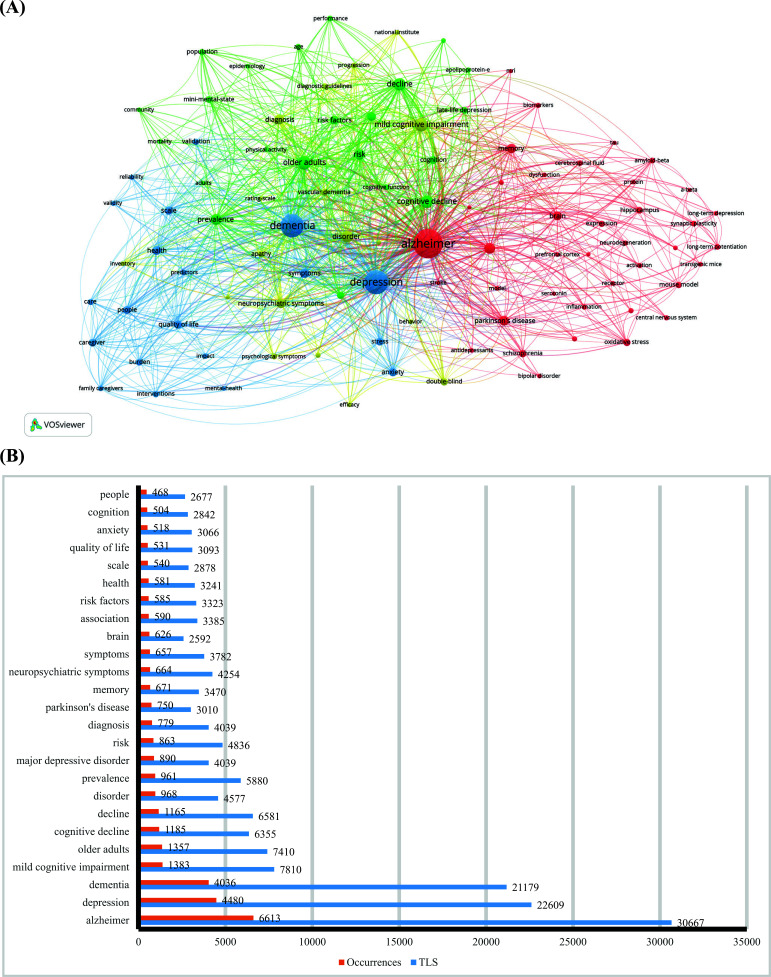
Analysis of keywords. (**A**) The co-occurrence networks of keywords are visualized by VOSviewer. Sizeable nodes denote phrases with high frequency, whilst nodes sharing the same colour indicate closer associations, (**B**) The top 25 keywords with the highest occurrences.

To visualize the frequently mentioned topics and scientific network, VOSviewer was employed to analyze a keyword co-occurrence. Moreover, a thesaurus was applied to merge similar keywords (Table **S1**). For example, depressive-like behaviour was replaced by depression and senile dementia was replaced by Alzheimer's. After the merging, 99 keywords with more than 150 occurrences are visualized on the map and are categorized into four clusters with various colours (Fig. **6A**). As shown in Fig. (**6B**), “Alzheimer” (n = 6613, TLS = 30667), “depression” (n = 4480, TLS = 22609) and “dementia” (n = 4036, TLS = 21179) were the keywords with the top 3 highest occurrences and TLS. We analysed the connections between the keywords in individual clusters and mapped them into sub-categories [48]. The content analysis of the keyword cluster landscape is shown in Fig. (**6A**). The most frequent keywords and sub-categories are presented in Table **5**. The theme analysis revealed four prevailing themes. The two largest themes are related to the mechanism between Alzheimer's disease and depression and cognitive decline in the elderly.

## 4 DISCUSSION

### 4.1 Analysis of Countries and Institutions

Almost all the top 10 contributing countries were developed countries, indicating that developed nations have recently given more consideration to mental health. The USA was predominant in the field, with 3881 publications and 238,425 total citations. Since AD increased age-standardized YLL rates in the USA, and major depressive disorder ranked 1^st^ in YLDs in 2010 [17], the social burden of AD and depression resulted in a significant number of publications in the USA. Furthermore, publications may also result from the challenge of ageing societies and substantial capital investment in scientific research. The United States Department of Health and Human Services, National Institutes of Health and NIA were the top three funders for this field, all from the USA [49]. China ranked second with 1003 publications and 24.9 citations per publication. Caregivers of individuals with AD experienced increased burden and worsened psychological states due to social isolation, physical inactivity, and sleep disturbance for at least 12 months after lockdown. Therefore, it is important to address these issues to improve the well-being of Chinese caregivers [50].

Most productive authors and institutions were from the USA (5/10 authors and 6/10 institutions), while none of them came from China, indicating that Chinese documents were distributed to many authors and institutions. The University of Pittsburgh, the University of California, Los Angeles and Johns Hopkins University collaborated, explaining why the publications of the USA led the head. In line with these publications, the USA ranked first in citations, demonstrating the high quality of these publications.

### 4.2 Analysis on Authors

New researchers could be provided with trendy themes and research concerns in identifying contributing authors' works. Lyketsos, Constantine G. and Cummings, Jl, the top 2 authors with high publications, came from the Johns Hopkins University (USA) and the University of Nevada (USA), respectively. Lyketsos, Constantine G. wrote 7 and 9 documents in 2013 and 2015, respectively, concentrating on the association among caregiver personality, depression, and AD, leading to the hotpots of the field [51, 52]. In 2021, Lyketsos, Constantine G. explored the association between two different kinds of symptoms, such as depression and postoperative delirium, hearing loss and neuropsychiatric symptoms among people with AD, suggesting that the currently popular theme was the link between different symptoms in AD [53, 54]. Lyketsos, Constantine G. was the center in collaboration with Brodaty, Henry, Schneider, Lon S., and Cummings, Jl, indicating the reason for his high citations. Cummings, Jl published 4 articles in 2007, focusing on the apathy of AD and the conversion from Mild Cognitive Impairment (MCI) subjects with depression to AD [55, 56].

### 4.3 Analysis on Journals

Only four journals among the top 10 productive journals were the journals with high citations, suggesting that the other six journals with high publications should improve the standard for publication. According to the Journal Citation Reports (JCR) in 2023, most of the top 10 co-cited journals were Q1 and Q2, indicating that these journals were generally recognized as high-quality forums. Neurology, Journal of Neuroscience and International Journal of Geriatric Psychiatry had co-citations over 10000, demonstrating that they had principal concerns about AD with depression. Therefore, reading classic articles in these journals with remarkable contributions could get familiar with differential scientific attention and the current hot topics.

### 4.4 Analysis on Keywords and Research Frontiers

A scientific network should be extensively evaluated before a high-quality scientific investigation is conducted. The analyses based on keywords and references should provide a comprehensive scientific network and visualize the interaction of different themes [12, 57]. By employing VOSviewer and CiteSpace, the scientific network of Alzheimer's disease with depression was visualized in terms of notable keywords and seminal references. The following section extensively evaluated the keywords and citations with cluster creation.

Cluster 1 (Red): The mechanism between Alzheimer's disease and depression

The primary keywords were “Alzheimer” “major depressive disorder”, “Parkinson's disease”, “memory,” and “brain”. There is a relationship between neuropsychiatric and neurodegenerative disorders. Mounting evidence shows that neuropsychiatric and neurodegenerative disorders have overlapping mechanisms. These mechanisms include signaling events, the microbiota-gut-brain axis, and post-translational modifications. There are various drug molecules with potential therapeutic effects in these disorders, including RNAs, multitarget-directed ligands, repurposed drugs, and natural compounds [58]. According to the bibliometric and scientometric analysis of AD in 2017, genetics is growing at the fastest pace, while pathophysiology and therapy have not progressed further. However, other research topics of growing interest include the role of microglia, inflammation, and synapses [59]. Consistent with this finding, the transcriptomic changes in brain areas play an important role. The perturbation of 19 genes in AD brain regions indicated the involvement of genes in AD neuropathology. The transcriptomic changes in AD brains might provide further insight into the underlying pathogenesis of AD [60]. The absence of gut microbiota caused changes in the transcriptome of microglial subpopulations, which were associated with AD and MDD, according to cross-species analysis and animal behavioral tests. Therefore, gut microbiota primarily regulates the transformation of microglial subtypes [61]. Many scientists focused on the synaptic plasticity in mouse models of Alzheimer's disease. Before the onset of plaque formation, the rise in extracellular matrix levels in hippocampal synaptosome preparations appeared. This increase was accompanied by impairments in hippocampal long-term potentiation and contextual memory, which suggest that the extracellular matrix plays a crucial role in causing early memory loss in AD [62]. The study identified a substrate for the synaptic disruption mechanism that underlies hippocampal cognitive deficits in the Aβ25-35 amyloidosis model of Alzheimer's disease [63]. Biomarkers in cerebrospinal fluid (CSF) are a significant theme. There were elevated levels of cerebrospinal fluid soluble amyloid protein procurer α (sAPPα) and β (sAPPβ) in individuals with pMCI, indicating that sAPPα and sAPPβ levels might act as indicators in the diagnosis of AD in early-stage [64]. In mild cognitive impairment patients with AD(-) biomarkers, white matter hyperintensities may play a role in cognitive impairment or depression [65]. Discovering the ameliorative role of drugs on neurodegenerative diseases is a popular topic. Lycopene has prophylactic and/or therapeutic effects in various types of disorders in the central nervous system, including AD, depression, cerebral ischemia, Huntington's disease (HD), Parkinson's disease (PD), and epilepsy [66].

Cluster 2 (Green): Cognitive decline and elderly

The primary keywords were “older adults”, “cognitive decline”, “decline”, “prevalence,” and “risk”. Meta-analysis mainly focused on cognitive function. The meta-analysis found a weak association between total sedentary time and cognitive function, which varies depending on how sedentary time is measured and which domain of cognitive function is being assessed [67]. The meta-analysis results showed that individuals with untreated hearing have significantly poorer cognition, and even after treatment, cognition remains poorer compared to those with normal hearing [68]. Late-life depression and cognitive decline in older adults is a significant issue. Managing depression in old patients with subjective cognitive decline was conducive to improving episodic memory, resulting in the delay of Alzheimer’s onset [69]. Loneliness had a positive correlation with the high risk of dementia in later life and a positive correlation with cognitive impairment and depression [70]. It was revealed that depression in the elderly increased the risk of AD [33]. Apolipoprotein-e might play a role in predicting cognitive decline. Depression and the apolipoprotein E ε4 allele had an association with memory impairment and reduced has-miR-107, a prominent risk marker for early AD [71]. A study suggests that APOE-e4 may be a predictor of heterogeneity in cognitive function, as it is linked to depression [72].

Cluster 3 (Blue): Characteristics, Burden of Care and Quality of Life in Dementia

The primary keywords were “depression”, “dementia”, “symptoms”, “health”, “scale”, and “quality of life”. Quality of life and depression in dementia is a major issue. According to a report, dementia patients living alone were much more likely to have insufficient self-care, falls, injuries, social isolation, missed or delayed diagnosis, unattended wandering, and death compared to patients living with others, and were much more likely to increase the payment for health care [73]. Anxiety and depression in caregivers of people with dementia is a problem to be resolved. Due to quarantine, behavioural and psychological symptoms (BPSD) saw a dramatic increase in 60% of patients and symptoms related to stress increased in two-thirds of caregivers. Depression and anxiety had a link with AD, especially in female patients with mild to moderate AD [74]. High burden, elevated anxiety, low social support, and young age in caregivers were regarded as the best indicators of unmet needs in individuals with dementia, indicating that caregivers should be given social support to improve the life quality of patients [75]. A lighting intervention targeting the circadian system had the ability to improve mood, sleep, and behavior in individuals with dementia [76]. Given the depression and quality of life in persons with dementia, the reliability and validity of scales are important. The internal consistency of the Portuguese version of Quality of Life-Alzheimer’s Disease (QOL-AD) was good for both caregiver and patient reports. The correlation of patient-reported QOL-AD with patient geriatric depression scale scores and satisfaction with life scale scores confirmed the construct validity. Thus, a Portuguese version of QOL-AD is proposed as a useful tool for research and clinical use [77].

Cluster 4 (Yellow): Meta-analysis on diagnosing and treating neuropsychiatric symptoms in patients with Alzheimer's disease

The primary keywords were “mild cognitive impairment”, “disorder”, “diagnosis”, and “neuropsychiatric symptoms”. Mild cognitive impairment and neuropsychiatric symptoms in Alzheimer’s disease are the scientists’ focus. The depressive symptom was a predictor for episodic memory impairment, so improving depression might prevent AD from cognitive impairment [78]. Due to the neuropsychiatric symptoms apparent in individuals suffering from amnestic Mild Cognitive Impairment (aMCI), their caregivers were experiencing emotional distress, similar to the pain experienced by caregivers of patients with AD. Therefore, it was highlighted that the emotional burden of caregivers should be reduced not only for their mental health but also for alleviating symptoms in patients with MCI or AD [79]. In patients with MCI, depression was found to be linked with a heightened risk of developing incident AD and thus was considered the earliest symptom of preclinical stages in AD. The treatment targeting neuropsychiatric symptoms was conducive to delaying the transition to dementia [80]. Furthermore, there is an association between psychological symptoms like apathy and the progression of dementia. Risk factors for worsening apathy include taking antidepressants, reduced inferior-temporal cortical thickness, longer duration of AD, being an ApoEε4 carrier, higher baseline apathy, lower cognitive test scores, higher midlife motivational abilities, and premorbid personality traits [81]. The pattern of symptoms, including apathy, agitation, disinhibition, irritability, and aberrant motor behaviour, worsens as the disease progresses in both early-onset and late-onset Alzheimer's disease [82].

## 5 LIMITATIONS

There are some limitations worth noting in this study. Firstly, because of the data format required for VOSviewer and CiteSpace, the manuscripts about Alzheimer's disease with depression were searched from a single database (WOSCC), leading to selection bias. As VOSviewer and CiteSpace were only compatible with some data sources, the other data sources were not used in this bibliometric analysis to reduce selection bias. Secondly, the exclusive consideration of literature in English led to language bias and lower credibility. Therefore, manuscripts in other languages should also be accepted in the future for rational analysis. Thirdly, although the employed search terms are based on Medical Subject Headings in NLM and the queries of other bibliometric analyses of Alzheimer's Disease or depression, the search terms are not comprehensive enough to encompass all relevant literature.

## CONCLUSION

The significance of depression in the development of Alzheimer's disease is increasing. Manuscripts investigating “Alzheimer”, “depression”, “dementia”, “mild cognitive impairment”, and “older adults” displayed a substantial number of citations per manuscript. Following are the research directions: (1) The exact mechanism of depression in AD should be explored to provide a theoretical basis for the therapy; (2) It is necessary to take more measures to alleviate the neuropsychiatric symptoms, especially depression, and improve the life quality of Alzheimer’s patients and their carers; (3) Identifying the cognitive decline and late-life depression in the Alzheimer’s disease is significant; (4) It is suggested to diagnose and treat neuropsychiatric symptoms in patients with Alzheimer's disease.

In recent years, the role of the mechanism in Alzheimer's disease and depression has been increasingly recognised. We have entered an era where it is not a question of whether people with Alzheimer's disease have neuropsychiatric symptoms, such as depression and low quality of life. Research in Alzheimer's disease is beginning to explore this area, with the hope of understanding the exact pathophysiology of depression in Alzheimer's disease. Our work presents a trend analysis of emerging issues related to Alzheimer's disease and depression. Some of them will not only remain in the coming years but may become the future for new mechanisms and a step forward in the ameliorative role of medicinal products. Neuropsychiatric disorders, such as depressive disorders, and neurodegenerative disorders, such as Alzheimer's disease, share several common mechanisms, including signalling events, the microbiota-gut-brain axis, and post-translational modifications. Therefore, studies that focus only on oxidative stress and synaptic plasticity may not be sufficient. Based on the findings of transcriptomic changes, there should be an increase in studies evaluating signalling pathways in cell and animal models and investigating whether depression in Alzheimer's disease can be treated with new drugs that target the exact signalling pathways.

In conclusion, our bibliometric analysis of the literature on Alzheimer's and depression shows clear trends in Alzheimer's and depression research, including publication, impact, study type, and topic. Our findings can help research funding agencies make informed decisions by highlighting past progress and identifying unresolved questions.

## Figures and Tables

**Fig. (2):**
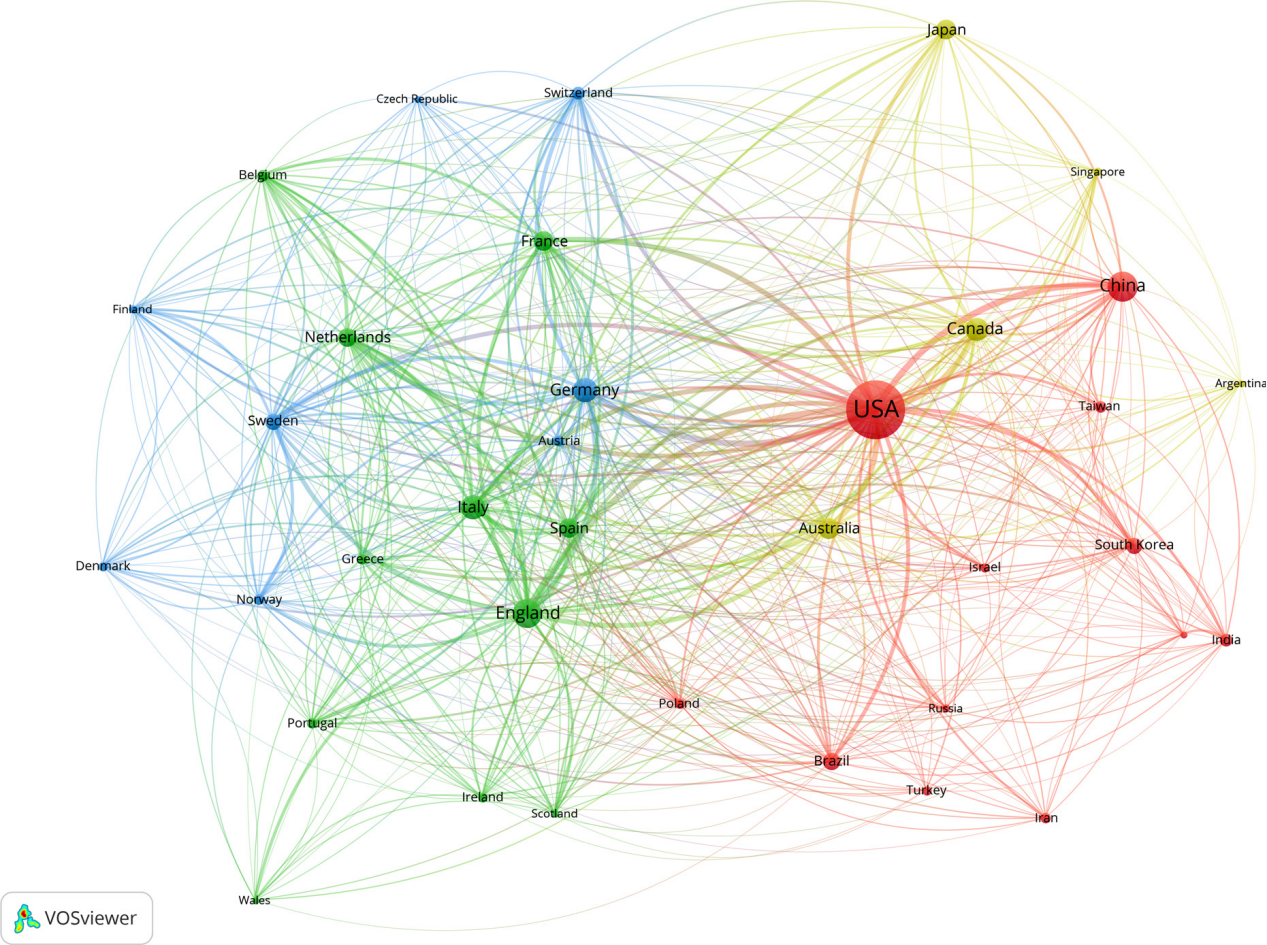
Cooperation between countries, with the size of nodes indicating the number of documents and the width of links representing collaboration strength.

**Fig. (3):**
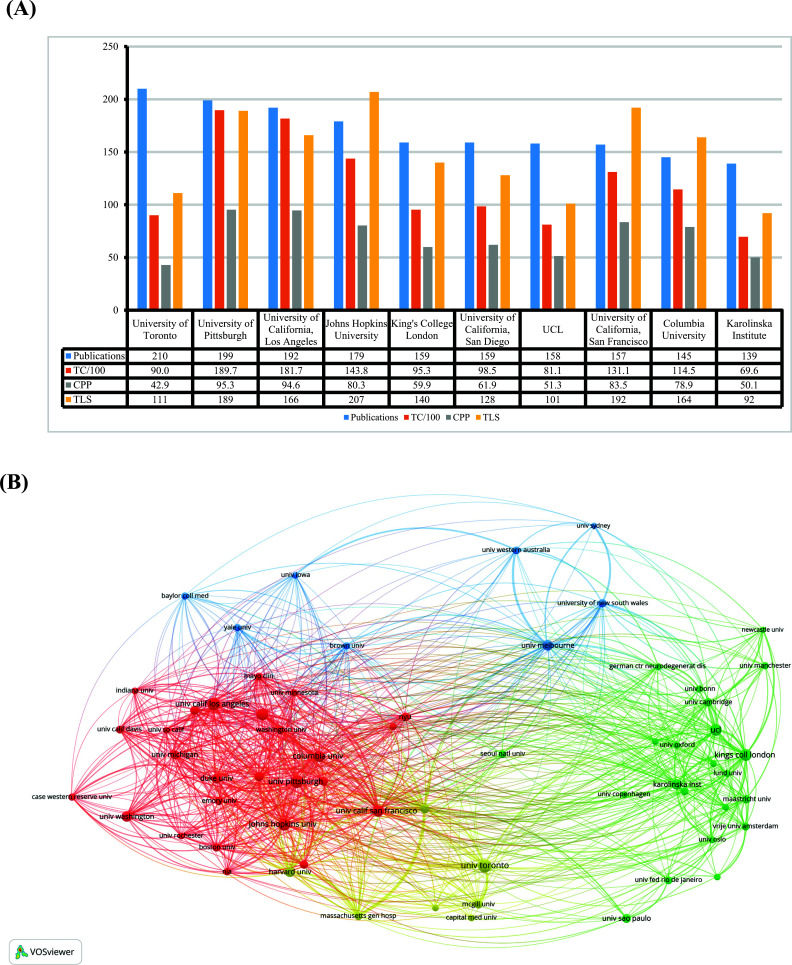
(**A**) Top 10 prolific institutions, the number of publications, total citations (×0.01), citations per publication and total link strength for each institution. (**B**) Institutions collaborating with each other. The size of the node shows how many documents they have. The thickness of the link shows how strong the cooperation is.

**Fig. (4):**
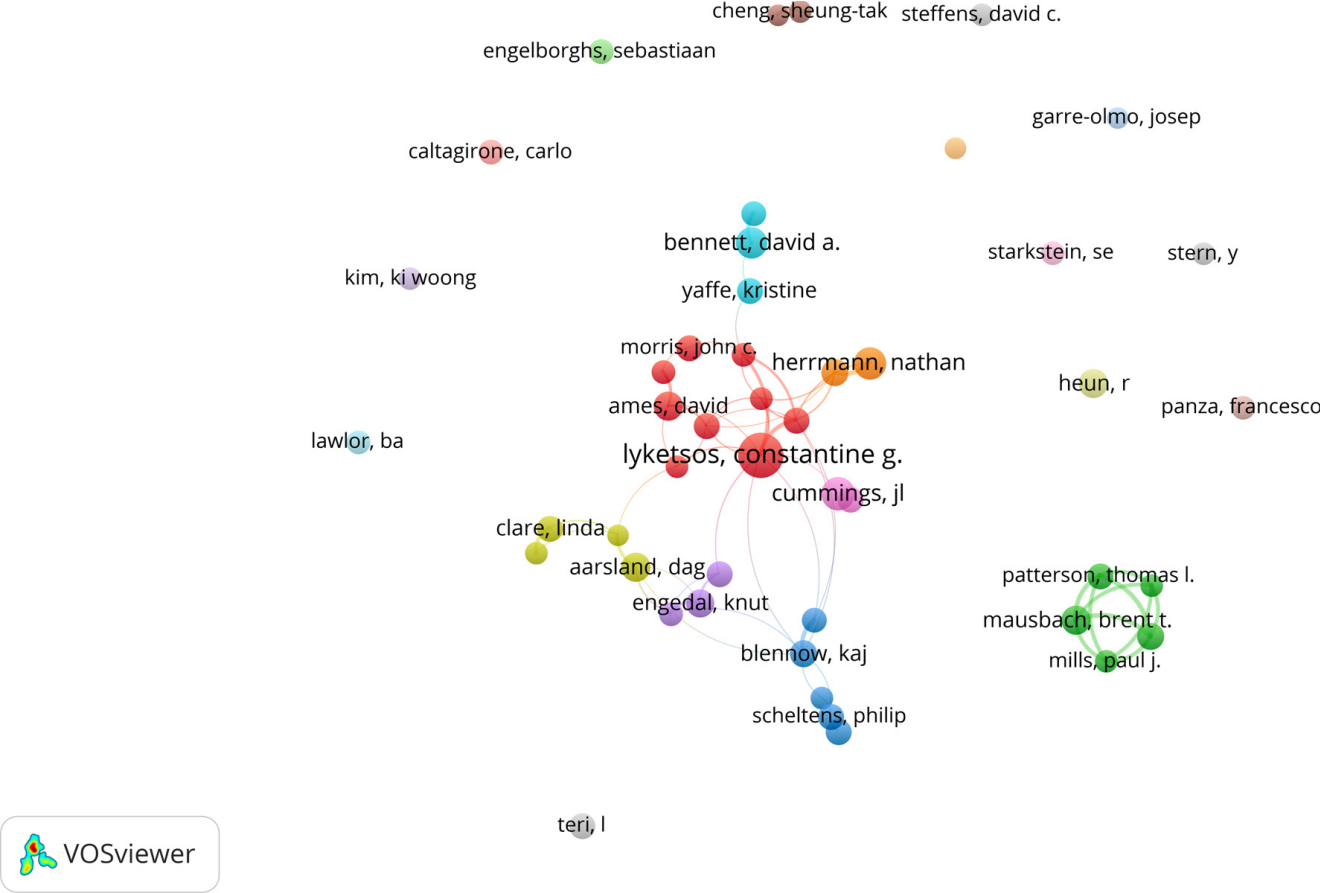
The network of collaboration between the most prolific authors, as shown by VOSviewer. The size of each node indicates how many documents they contributed to, while the width of the links displays the strength of their collaboration.

**Table 1:** Top 10 prolific countries, the number of documents, total citations, citations per publication, total link strength, and links for each country/region.

**Rank**	**Country/Region**	**Publications**	**TC**	**CPP**	**TLS**	**Links**
1	USA	3881	238425	61.4	2009	87
2	China	1003	24940	24.9	448	54
3	England	951	58242	61.2	1242	69
4	Germany	704	36198	51.4	901	65
5	Italy	663	28265	42.6	693	54
6	Canada	640	35440	55.4	578	49
7	Australia	525	30371	57.8	625	56
8	France	465	24550	52.8	614	57
9	Spain	442	16695	37.8	560	56
10	Japan	438	14358	32.8	238	46

**Table 2:** The top 11 prolific authors and co-cited authors.

**Rank**	**Author**	**Publication**	**TC^1^**	**CPP^2^**	**TLS**	**Institution**	**Country/ Region**	**Rank**	**Co-cited Author**	**Co-citation**	**Institution**	**Country/ Region**
1	Lyketsos, Constantine G.	84	9598	114.3	48	Johns Hopkins University	USA	1	Folstein, Mf	2721	Tufts University	USA
2	Cummings, Jl	47	8302	176.6	2	University of Nevada	USA	2	Cummings, Jl	2097	University of Nevada	USA
3	Herrmann, Nathan	42	1325	31.5	30	University of Toronto	Canada	3	Mckhann, G	1948	Columbia University	USA
4	Bennett, David A.	41	3406	83.1	23	Rush University	USA	4	Lyketsos Constantine, G	1530	Johns Hopkins University	USA
5	Aarsland, Dag	36	1268	35.2	18	King's College London	England	5	Alexopoulos, Gs	1338	Cornell University	USA
6a	Ames, David	34	2628	77.3	22	University of Melbourne	Australia	6	Petersen, Rc	1312	Mayo Clinic	USA
6b	Heun, R	34	1182	34.8	0	University of Birmingham	England	7	Morris, Jc	1158	University of Washington	USA
6c	Mausbach, Brent T.	34	1304	38.4	98	University of California San Diego	USA	8	Starkstein, Se	1137	University of Western Australia	Australia
9	Engedal, Knut	32	995	31.1	26	University of Oslo	Norway	9	Wilson, Rs	1076	Rush University	USA
10a	Grant, Igor	31	1164	37.5	98	University of California San Diego	USA	10	Teri, L	983	University of Washington	USA
10b	Lanctot, Krista L.	31	1394	45.0	35	Univ Toronto	Canada	11	Reisberg, B	974	NYU	USA

**Note: **^1^Total citations. ^2^Citations per publication.

**Table 3:** Top 10 cited articles.

**Rank**	**Title**	**First author**	**Type**	**Journal**	**Year**	**Citations**
1	Mechanisms and Functional Implications of Adult Neurogenesis [16]	Zhao, Chunmei	Review	Cell	2008	2342
2	The Projected Effect of Risk Factor Reduction on Alzheimer's Disease Prevalence [18]	Barnes, Deborah E.	Review	Lancet Neurology	2011	1768
3	The State of US Health, 1990-2010 Burden of Diseases, Injuries, and Risk Factors [17]	Murray, Christopher J. L.	Article	Jama-Journal of the American Medical Association	2013	1679
4	Global, Regional, and National Incidence, Prevalence, and Years Lived with Disability for 328 Diseases and Injuries for 195 Countries, 1990-2016: A Systematic Analysis for The Global Burden of Disease Study 2016 [15]	Vos, Theo	Article	Lancet	2017	1521
5	Potential for Primary Prevention of Alzheimer's Disease: An Analysis of Population-Based Data [21]	Norton, Sam	Article	Lancet Neurology	2014	1501
6	The Neuropsychiatric Inventory: Assessing Psychopathology in Dementia Patients [19]	Cummings, Jl	Review	Neurology	1997	1481
7	Prevalence of Neuropsychiatric Symptoms in Dementia and Mild Cognitive Impairment - Results from The Cardiovascular Health Study [20]	Lyketsos, Cg	Article	Jama-Journal of the American Medical Association	2002	1418
8	The Role of the Posterior Cingulate Cortex in Cognition and Disease [23]	Leech, Robert	Review	Brain	2014	1389
9	The Spectrum of Disease in Chronic Traumatic Encephalopathy [22]	Mckee, Ann C.	Article	Brain	2013	1361
10	Metabotropic Glutamate Receptors: Physiology, Pharmacology, and Disease [24]	Niswender, Colleen M.	Review	Annual Review of Pharmacology and Toxicology	2010	1282

**Table 4:** The top 10 prolific journals and co-cited journals.

**Rank**	**Journal**	**Publications**	**TC^1^**	**CPP^2^**	**TLS**	**IF^3^ (2023)**	**JCR^4^ (2023)**	**Co-cited Journal**	**Co-citations**	**IF (2023)**	**JCR (2023)**
1	Journal of Alzheimer's Disease	505	13500	26.7	3426	4.0	Q2	Neurology	20721	9.9	Q1
2	International Journal of Geriatric Psychiatry	407	15862	39.0	3674	4.0	Q2	Journal of Neuroscience	11956	5.3	Q1
3	American Journal of Geriatric Psychiatry	251	13026	51.9	2958	7.2(2022)	Q1(2022)	International Journal of Geriatric Psychiatry	10300	4.0	Q2
4	International Psychogeriatrics	213	7593	35.6	1731	7.0	Q1	American Journal of Psychiatry	9630	17.7	Q1
5	Dementia and Geriatric Cognitive Disorders	176	8029	45.6	1701	2.4	Q3	Proceedings of the National Academy of Sciences of the United States of America	8977	11.1	Q1
6	Aging and Mental Health	152	4402	29.0	840	3.4	Q2	Journal of Alzheimer's Disease	8650	4.0	Q2
7	Alzheimer's Disease and Associated Disorders	133	4667	35.1	1034	2.1	Q3	Archives of Neurology (Jama Neurology)	8243	29.0	Q1
8	Journal of Geriatric Psychiatry and Neurology	123	3338	27.1	1038	2.6	Q3	Biological Psychiatry	8064	10.6	Q1
9	Journal of the American Geriatrics Society	117	9750	83.3	972	6.3	Q1	Journal of the American Geriatrics Society	7853	6.3	Q1
10	Frontiers in Aging Neuroscience	116	2823	24.3	587	4.8	Q1	American Journal of Geriatric Psychiatry	7471	7.2 (2022)	Q1 (2022)

**Note: **^1^Total citations, ^2^Citations per publication, ^3^Impact factor, ^4^Journal Citation Reports.

**Table 5:** Small-size sample research theme.

**Theme**	**Colour**	**More Frequent Keywords**	**Prevailing Sub-categories**
The mechanism between Alzheimer's disease and depression	Red	Alzheimer’s (6613), major depressive disorder (890), Parkinson's disease (750), memory (671), brain (626), schizophrenia (421), oxidative stress (394), hippocampus (338), expression (327), mouse model (293), synaptic plasticity (282), long-term potentiation (279), amyloid-beta (274), cerebrospinal fluid (267), neurodegeneration (237), inflammation (231), biomarkers (226),	Relationship between neuropsychiatric and neurodegenerative disorders; oxidative stress in mice with Alzheimer's and depression; transcriptomic changes in brain areas, synaptic plasticity in mouse models of Alzheimer's; cerebrospinal fluid biomarkers; ameliorative role of drugs on neurodegenerative diseases
Cognitive decline and elderly	Green	Older adults (1357), cognitive decline (1185), decline (1165), prevalence (961), risk (863), association (590), risk factors (585), cognition (504), meta-analysis (429), late-life depression (364), mini-mental-state (345), population (328), performance (282), age (274), cognitive function (250), apolipoprotein-e (232)	Meta-analysis on cognitive function, late-life depression and cognitive decline in older adults, apolipoprotein-e predicts cognitive decline
Characteristics, burden of care and quality of life in dementia	Blue	Depression (4480), dementia (4036), symptoms (657), health (581), scale (540), quality of life (531), anxiety (518), people (468), stress (457), caregiver (453), burden (378), interventions (318), validation (301), care (289), family caregivers (285), predictors (270), impact (242), reliability (231), validity (220)	Quality of life and depression in dementia, interventions, anxiety and depression in caregivers of people with dementia, reliability and validity of scales in persons with dementia
Diagnosing and treating neuropsychiatric symptoms in Alzheimer's disease	Yellow	Mild cognitive impairment (1383), disorder (968), diagnosis (779), neuropsychiatric symptoms (664), double-blind (431), vascular dementia (381), apathy (300), psychological symptoms (279), diagnostic guidelines (275), psychosis (226), progression (209)	Mild cognitive impairment and neuropsychiatric symptoms in Alzheimer’s disease, association between psychological symptoms like apathy and progression in dementia

**Note:** Numbers in parenthesis present the number of papers in which a keyword occurred.

## Data Availability

Not applicable.

## LIST OF ABBREVIATIONS

aMCI: Amnestic Mild Cognitive Impairment
BPSD: Behavioural and Psychological Symptoms
CSF: Cerebrospinal Fluid
GMV: Gray Matter Volume
HD: Huntington's Disease
MCI: Mild Cognitive Impairment
MDD: Major Depressive Disorder
PD: Parkinson's Disease
SGZ: Subgranular Zone
SVZ: Subventricular Zone
