# Supplementary material for: Bibliometric Analysis of Alzheimer's Disease and Depression
Source: Curr Neuropharmacol. 2024 Jul 31;23(1):98–115. doi: 10.2174/1570159X22666240730154834 (PMC11519817; doi:10.2174/1570159X22666240730154834)
Supplement: Supplementary file 1 [file CN-23-98_SD1.pdf]

Supplementary Material

Bibliometric Analysis of Alzheimer's Disease and Depression

Sixin Li[1,2,#], Qian Zhang[3,4,#], Jian Liu[5], Nan Zhang[6], Xinyu Li[1,2], Ying Liu[1,2], Huiwen Qiu[1,2], Jing Li[7,*] and Hui Cao[1,2,*]

[1]Department of Psychiatry, The School of Clinical Medicine, Hunan University of Chinese Medicine, Changsha, Hunan, China; [2]Department of Psychiatry, Brain Hospital of Hunan Province (The Second People's Hospital of Hunan Province), Changsha, Hunan, China; [3]Department of Neurosurgery, Xiangya Hospital, Central South University, Changsha, Hunan, China; [4]National Clinical Research Center for Geriatric Disorders, Xiangya Hospital, Central South University, Hunan, China; [5]Center for Medical Research and Innovation, The First Hospital, Hunan University of Chinese Medicine, Changsha, Hunan, China; [6]College of Life Science and Technology, Huazhong University of Science and Technology, Wuhan, Hubei, 430074, P.R.China; [7]Department of Rehabilitation, The Second Xiangya Hospital, Central South University, Changsha, Hunan, China

1875-6190/25 $65.00+.00 **© 2025 Bentham Science Publishers**

Table S1. Thesaurus.

Label	Replace by
Senile dementia	Alzheimer
Alzheimer disease	Alzheimer
Alzheimer&#8217	Alzheimer
Alzheimer's	Alzheimer
Alzheimer's disease	Alzheimer
Alzheimer's disease (ad)	Alzheimer
Alzheimer-disease	Alzheimer
Alzheimers	Alzheimer
Alzheimers association workgroups	Alzheimer
Alzheimers disease	Alzheimer
Alzheimers-disease	Alzheimer
Ad	Alzheimer
Carers	Caregiver
Caregivers	Caregiver
Cognitive deficits	Cognitive decline
Cognitive dysfunction	Cognitive decline
Cognitive impairment	Cognitive decline
Impairment	Decline
Deficits	Decline
Memory deficits	Memory impairment
Major depression	Major depressive disorder
Depressive symptoms	Depression
Depressive-like behavior	Depression
Disease	Disorder
Disorders	Disorder
Metaanalysis	Meta-analysis
Mini-mental state	Mini-mental-state
Aging	Older adults
Aged	Older adults
Older	Older adults
Older persons	Older adults
Older-adults	Older adults
Older-people	Older adults
Elderly	Older adults
Elderly-people	Older adults

**Label**	**Replace by**
Parkinsons-disease	Parkinson's disease
Quality-of-life	Quality of life
Risk factor	Risk factors
Risk-factor	Risk factors
Risk-factors	Risk factors
Antidepressant	Antidepressants
Apolipoprotein e	Apolipoprotein-e
Biomarker	Biomarkers
Central-nervous-system	Central nervous system
Cerebral blood-flow	Cerebral-blood-flow
Cerebrospinal-fluid	Cerebrospinal fluid
Huntingtons-disease	Huntington's disease
Intervention	Interventions
Mental health	Mental-health
Multiple sclerosis	Multiple-sclerosis
Neurodegenerative diseases	Neurodegenerative disease
Neurodegenerative disorders	Neurodegenerative disease
Nursing-home	Nursing home
Physical-activity	Physical activity
Positron-emission-tomography	Positron emission tomography
Randomized controlled-trial	Randomized controlled trial
Receptors	Receptor
Traumatic brain-injury	Traumatic brain injury
Transgenic mouse model	Transgenic mice
Traumatic brain-injury	Traumatic brain injury
Diabetes-mellitus	Diabetes
Diagnostic-criteria	Diagnostic guidelines
Mci	Mild cognitive impairment
Psychiatric-disorders	Psychiatric disorders
